# *Motilimonas cestriensis* sp. nov., isolated from an inland brine spring in Northern England

**DOI:** 10.1099/ijsem.0.004763

**Published:** 2021-03-19

**Authors:** Matthew Kelbrick, Raeid M. M. Abed, André Antunes

**Affiliations:** ^1^​ Biology Department, Edge Hill University, Ormskirk, UK; ^2^​ Department of Evolution, Ecology and Behaviour, Institute of Infection, Veterinary & Ecological Sciences, University of Liverpool, Liverpool, UK; ^3^​ Biology Department, College of Science, Sultan Qaboos University, Al Khoud, Sultanate of Oman; ^4^​ State Key Laboratory of Lunar and Planetary Sciences, Macau University of Science and Technology (MUST), Macau SAR, PR China; ^5^​ China National Space Administration (CNSA), Macau Center for Space Exploration and Science, Macau SAR, PR China

**Keywords:** *Motilimonas cestriensis *sp. nov., halophilic bacteria, brine spring

## Abstract

A novel slightly halophilic Gram-stain-negative bacterial strain (MKS20^T^) was isolated from a brine sample collected from one of the Anderton brine springs in the Cheshire salt district, located in Northern England. Phylogenetic analysis of the 16S rRNA gene sequence revealed a close proximity to *Motilimonas eburnea* (98.30 %), followed by *Motilimonas pumila* (96.62 %), the two currently described species within the genus *Motilimonas*. Strain MKS20^T^ forms white-beige-pigmented colonies and grows optimally at 28–30 °C, in 1–3 % (w/v) NaCl and at pH 7–7.5. The strain was facultatively anaerobic and showed a broader range of carbohydrate use than other species in the genus *Motilimonas*. Q-8 was the sole respiratory quinone and the major fatty acids (>10 %) were summed feature 3 (C_16 : 1_ ω6*c* and/or C_16 : 1_ ω7*c*) and C_16 : 0_. The polar lipid profile included diphosphatidylglycerol, phosphatidylethanolamine, phosphatidyglycerol and several unidentified lipids. The G+C content of the genomic DNA was 44.2 mol%. Average nucleotide identity and DNA–DNA hybridization data were consistent with assignment to a separate species. Based on the phylogenetic and genomic-based analyses, as well as physiological and biochemical characteristics, we propose that strain MKS20^T^ (=DSM 109936^T^, MCCC 1K04071^T^) represents a new species of the genus *Motilimonas*, with the name *Motilimonas cestriensis* sp. nov.

## Introduction

The Cheshire salt district (UK) has an industrial heritage of salt production dating back to the Romans (first–second centuries AD) who excavated the land for its abundant salt-rock [1]. This industry was enabled by the Cheshire salt district’s location over subterranean Triassic salt deposits, which are currently still quarried in the Winsford salt mine and extracted via brine pumping at the Halford brine fields. The deposits have also given rise to a few natural saline biotopes such as those present in Anderton, where surface-derived groundwater is in contact with salt-rock, forming brine which springs to the surface [1]. This process originates multiple interlocking brine pools which seasonally fluctuate in salinity (1.3–13 % NaCl, w/v) due to variation in rainfall, evaporation and drainage.

Saline biotopes such as these are inhabited by uniquely adapted halophilic and/or halotolerant micro-organisms, and thus have become sought-after for their taxonomic novelty and potential biotechnological applications [2]. Despite this, the numerous saline biotopes of the Cheshire salt district have undergone very limited microbiological studies, with most focusing only on the Winsford salt mine [3, 4].

During an astrobiological- and bioprospection-based study of the Anderton brine springs, several microbial strains were isolated [5]. The majority of the strains were assigned to already known species of the genera *Bacillus*, *Halomonas*, *Salinivibrio*, *Pseudoalteromonas*, *Marinobacter* and *Planococcus* and many showed production of hydrolases and anti-microbial compounds. Noteworthy among these new isolates was a novel white-beige pigmented bacterial strain, designated MKS20^T^. The taxonomic position of strain MKS20^T^ was investigated using 16S rRNA gene sequencing, with the results indicating that it was affiliated to *Motilimonas*, a genus *incertae sedis* within the order *Alteromonadales* (www.bacterio.net/-classifphyla.html) [6]. At the point of publication, the only two validly described species of this genus are *Motilimonas eburnea*, which was isolated from a marine sediment in China, and *Motilimonas pumila*, which was isolated from the gut of the sea cucumber *Apostichopus japonicus* [7, 8]. Based on polyphasic characterization we showed that strain MKS20^T^ could be distinguished from the other species within the genus *Motilimonas*. We therefore propose the name *Motilimonas cestriensis* sp. nov. for the species represented by strain MKS20^T^.

Strain MKS20^T^ was isolated from a brine sample collected from the Anderton brine springs, Cheshire, UK (53° 16′ 19.7″ N, 2° 31′ 28.0″ W), which, at the time of sampling, had a salinity of 4.2 %, a pH of 7.4 and temperature of 14 °C. Aliquots of the sample were serially diluted and streaked on nutrient agar (NA; Oxoid) supplemented with 5 % (w/v) NaCl (Melford) and incubated at 30 °C for 3 days. A white-beige circular colony was observed and purified via subculturing on the same medium before being preserved at −80 °C in nutrient broth (NB; Oxoid) supplemented with 2 % (w/v) NaCl and 20 % (v/v) glycerol (Melford). After purification, standard cultivation was done on NA with salinity adjusted to 2 % (w/v) NaCl to reduce osmotic stress and increase growth rate. The type strains, *M. eburnea* YH6^T^ (MCCC 1H00122^T^) and *M. pumila* PLHSC7-2^T^ (MCCC 1K03522^T^), were obtained from the Marine Culture Collection of China (MCCC). For all experiments, the three strains were grown on NA supplemented with 2 % (w/v) NaCl at pH 7.5 and incubated at 30 °C, unless otherwise stated.

Genomic DNA of strain MKS20^T^ was extracted using Griffith’s method [9]. PCR amplification of the 16S rRNA gene was performed using the forward primer 27F (Sigma; 5′- AGAGTTTGATCCTGGCTCAG-3′) and the reverse primer 1492R (Sigma; 5′-TACCTTGTTACGACTT-3′) [10]. PCR products were purified using the Sigma-Aldrich GenElute PCR Clean-Up Kit according to the manufacturer’s instructions, and sent to Eurofins for Sanger sequencing (Konstanz). The 16S rRNA gene similarity with published relatives was examined using NCBI blast (www.ncbi.nlm.nih.gov) and EzTaxon-e (www.ezbiocloud.net) [11]. The genomic DNA of MKS20^T^ and *M. eburnea* was extracted and sequenced by MicrobesNG (Birmingham, UK) using Illumina HiSeq 2500 apparatus with the 250 bp paired-end protocol and a minimum coverage of 30×. The Bioinformatics services of MicrobesNG trimmed the DNA reads using Trimmomatic 0.30 [12], performed a *de novo* assembly using SPAdes version 3.8 [13], and annotated the contigs using Prokka [14]. Genome sequences were checked for contamination using ContEst16S (www.ezbiocloud.net/tools/contest16s) [15], and the 16S rRNA gene sequence of strain MKS20^T^ (accession number: MW130885) and *M. eburnea* (accession number: KR610526) obtained via Sanger sequenced were compared with the 16S rRNA gene from their respective genome sequence using NCBI blast to confirm genome authenticity before deposition via the services of GFBio [16]. The genome of *M. pumila* PLHSC7-2^T^ was obtained from NCBI (accession: QZCH00000000) [8] and its DNA had a G+C content of 45.5 mol%. The genome size of MKS20^T^ was 4 796 511 bp, and the G+C content was 44.2 mol% (ENA accession: PRJEB39676). The genome was composed of 168 contigs with N50 and L50 values of 170 736 nt and 9, respectively. In comparison, the genome size of *M. eburnea* was 4 618 837 bp and the G+C content was 46.2 mol% (ENA accession: PRJEB39823). The genome was composed of 189 contigs with an N50 of 160 712 nt and an L50 of 10.

All genomes were annotated using the rast Annotation Server (http://rast.theseed.org). Analysis of annotated subsystem features indicated the presence of features in strain MKS20^T^ that were absent in *M. eburnea* and/or *M. pumila*. These included subsystems features of capsular and extracellular polysaccharides, osmotic regulation (ectoine biosynthesis), detoxification (formaldehyde), metabolism of aromatic compounds (benzoate degradation) and carbohydrate metabolism, which corresponded with strain MKS20^T^’s wider metabolic range as observed experimentally (see Table 1).

**Table 1.** Physiological and biochemical characteristics which differentiate strain MKS20^T^ from *M. eburnea* YH6^T^ and/or *M. pumila* PLHSC7-2^T^ All strains were negative for β-galactosidase, arginine dihydrolase, lysine decarboxylase, ornithine decarboxylase, H_2_S production, urease, tryptophane deaminase, indole production, gelatinase and Voges–Proskauer test. All strains were positive for acid production from d-glucose, d-fructose, d-mannose, *N*-acetyl-glucosamine and maltose, but negative for erythritol, d-arabinose, l-arabinose, d-ribose, d-xylose, l-xylose, d-adonitol, methyl β-d-xylopyranoside, d-galactose, l-sorbose, l-rhamnose, dulcitol, inositol, d-sorbitol, methyl α-d-mannopyranoside, methyl α-d-glucopyranoside, amygdalin, arbutin, aesculin ferric citrate, salicin, cellobiose, lactose, melibiose, sucrose, inulin, melezitose, raffinose, starch, glycogen, xylitol, gentiobiose, turanose, d-tagatose, d-fucose, l-fucose, d-arabitol, l-arabitol, potassium gluconate and potassium 2-ketogluconate. Cells tested positive for the enzymes esterase lipase (C8), leucine arylamidase and naphthol-AS-BI-phosphohydrolase, but negative for lipase (C14), valine arylamidase, cystine arylamidase, trypsin, α-chymotrypsin, α-galactosidase, β-galactosidase, β-glucuronidase, α-mannosidase, α-fucosidase and β-glucosidase. All strains were able to oxidise cellobiose, d-fructose, *N*-acetyl-d-galactosamine, α-d-glucose, d-mannose, *N*-acetyl-d-glucosamine and acetoacetic acid, but were unable to oxidise sucrose, stachyose, raffinose, lactose, *N*-acetyl neuraminic acid, d-salicin, melibiose, d-galactose, 3-methyl glucose, d-fucose, l-fucose, l-rhamnose, d-arabitol, γ-amino-butryric acid, d-glucose-6-PO_4_, d-fructose-6-PO_4_, d-aspartic acid, glycyl-l-proline, l-arginine, l-pyroglutamic acid, pectin, d-galacturonic acid, l-galactonic acid lactone, d-gluconic acid, d-glucuronic acid, mucic acid, quinic acid, d-saccharic acid, p-hydroxy-phenylacetic acid, d-lactic acid methyl ester, l-lactic acid, citric acid, α-keto-glutaric acid, d-malic acid, myo-inositol, α-hydroxybutyric acid, β-hydroxy-d,l-butyric acid, α-keto-butyric acid and formic acid.

Characteristics	MKS20^T^	*M. eburnea* YH6^T^	*M. pumila* PLHSC7-2^T^
Source of isolation	Inland brine spring	Marine sediment	Gut of sea cucumber
Cell Size (µm)	0.5–1.0 x 1.3–3.0	0.3–0.9 x 1.1–2.4	0.5–0.9 x 1.0–2.2
Growth at/with:			
Temperature (°C)	5–37	15–37	10–37
NaCl (%, w/v)	0.5–10.0	1–8	1–8
pH	6.0–9.5	6.5–9.0	6.5–8.5
Enzymatic activity:			
Alkaline phosphatase	+	−	+
Esterase (C4)	+	−	+
Acid phosphatase	+	−	+
α-Glucosidase	+	−	−
Amylase	−	−	+
Catalase	+	−	+
*N*-Acetyl-β-glucosaminidase	+	−	+
Acid production from:			
Glycerol	+	−	−
d-Mannitol	+	−	−
Trehalose	+	−	−
Potassium 5-ketogluconate	−	+	+
Oxidation of:			
Dextrin	+	−	−
Maltose	+	−	+
Trehalose	+	−	−
Gentiobiose	+	−	−
Turanose	+	−	−
Propionic acid	−	−	+
*N*-Acetyl-β-d-mannosamine	+	−	+
Inosine	+	+	−
d-Sorbitol	+	−	+
d-Mannitol	+	−	−
Glycerol	+	−	−
d-Serine	−	+	−
l-Alanine	+	−	−
l-Aspartic acid	+	−	−
l-Glutamic acid	+	−	−
l-Histidine	−	−	+
l-Serine	+	−	−
Glucuronamide	−	−	+
Methyl pyruvate	+	−	+
l-Malic acid	+	−	+
Bromo-succinic acid	+	−	−
Tween 40	+	−	+
Methyl β-d-glucoside	−	−	+
Acetic acid	+	−	+

Digital DNA–DNA hybridization (dDDH) was performed using DSMZ’s Genome-to-Genome Distance Calculator (GGDC; http://ggdc.dsmz.de/), applying statistical ‘formula two’ to calculate high-41 scoring segment pairs and sequence identities [17, 18]. Average nucleotide identity (ANI) and average amino-acid identity (AAI) was calculated using Kostas lab’s bioinformatic tools (http://enve-omics.ce.gatech.edu/) [19].

**Fig. 1.**
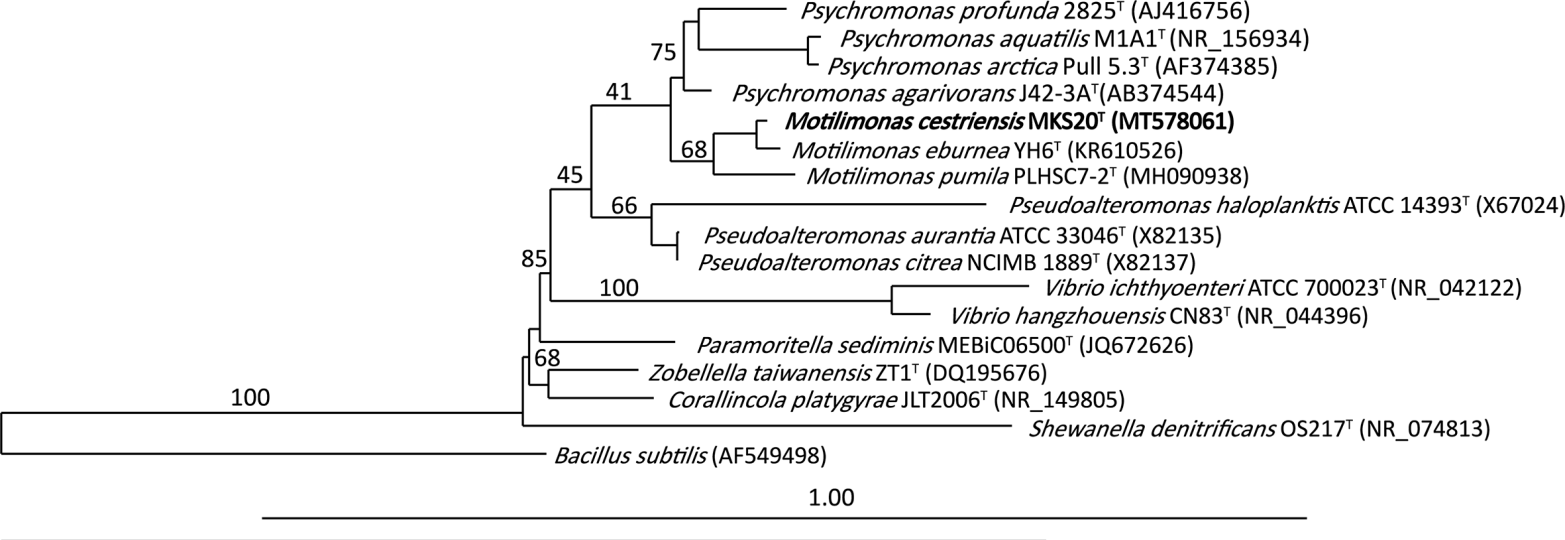
Maximum-likelihood phylogenetic tree based on the 16S rRNA gene sequences of strain MKS20^T^, *M. eburnea*, *M. pumila* and its closely related species, with bootstrap values being represented as a percentage.

The full 16S rRNA gene sequence of MKS20^T^ was extracted from the genomic DNA, the 16S rRNA gene length of MKS20^T^ was 1585 bp (accession number: MT578061). This sequence, combined with those of *M. pumila* and *M. eburnea*, were used to recalculate the 16S rRNA gene sequence relatedness between the three strains of *Motilimonas* using NCBI blastn. arb-silva (www.arb-silva.de) [20] was used to reconstruct a maximum-likelihood phylogenetic tree supplemented with selected type strains from NCBI that showed close relatedness to strain MKS20^T^ (Fig. 1). ModelTest was performed to select the best-fit nucleotide substitution model [21, 22] using the open-source software PhyML 3.0 [23] (www.atgc-montpellier.fr/phyml/). The phylogenetic tree was then re-calculated using the best substitution model GTR+G+IT. The robustness of the phylogenetic tree was confirmed by comparing this tree with all trees calculated with arb using all available models.

The 16S rRNA gene similarity between MKS20^T^ and *M. pumila* was 96.62 %, and the similarity between MKS20^T^ and *M. eburnea* was 98.30 %, which is lower than the 98.7 % species threshold set by Stackebrandt and Eders [24]. In addition, the low dDDH of <70 % supports that all three strains of *Motilimonas* are indeed different species, according to the guidelines set by Meier-Kolthoff [18] (Table S1, available in the online version of this article). Furthermore, the G+C content of the computed genome sequences of strain MKS20^T^, *M. eburnea* and *M. pumila* was 44.2, 46.2, and 45.5 mol% respectively, the >1 mol% variation between strain MKS20^T^ and the other two species is indicative of strain MKS20^T^ belonging to a separate species [18]. This is further confirmed by the ANI and AAI similarity values (Table S1), which hold the novel species thresholds of <95 % and <85 % respectively [25] (Table S1).

For phenotypic characterization, all three strains were grown on NA (or NB for growth experiments) supplemented with 2 % (w/v) NaCl, at pH 7.5, and incubated at 30 °C, unless otherwise stated. Cell morphology was examined after Gram-staining under a light microscope at ×1000 magnification succeeding calibration with a 100 µm microscope ruler. Cell motility was tested using the hanging drop method. Growth ranges and optima were measured via optical density in NB at a salinity of 0, 0.5, 1, 2, 3, 4, 5, 8, 10 and 12 % (w/v) NaCl; temperatures of 5, 10, 15, 20, 25, 28, 30, 33, 35, 37 and 40 °C; and at pH 5.5, 6.0, 6.5, 7.0, 7.5, 8.0, 8.5, 9.0, 9.5 and 10.0. The media for pH experiments were buffered using 50 mM MES (pH 5.5–6.5; Sigma-Aldrich), HEPES (pH 7.0–7.5; Sigma-Aldrich), TAPS (pH 8.0–8.5; Sigma-Aldrich) or CAPSO (pH 9.0–10.0; Sigma-Aldrich) [26]. The potential for anaerobic growth was examined on NA with and without 0.1 % NaNO_3_ (Sigma-Aldrich) for 14 days in an anaerobic jar containing a bioMérieux GenBag and anaerobic indicator strip.

Carbon source oxidation was determined using Biolog GEN III MicroPlates inoculated using Biolog Inoculation Fluid-A, supplemented with 2 % (w/v) NaCl, and incubated for 3 days following the manufacturer’s instructions. Carbon source acidification was determined using API 50CH test strips (bioMérieux), inoculated using 50 CHB/E medium (bioMérieux) supplemented with 2 % (w/v) NaCl and incubated for 3 days. Enzymatic and biochemical properties of the strains were analysed using API 20E and API ZYM test strips (bioMérieux), according to the manufacture’s protocols, with the modification of the inoculum being prepared with a 2 % (w/v) saline solution. Nitrate reduction was also tested as part of the API 20E test kit. The presence of catalase was tested by suspending a 48 h grown culture in a drop of hydrogen peroxide (Sigma-Aldrich), while the presence of oxidase was tested using a 24 h culture and one drop of oxidase reagent (bioMérieux). Amylase production was tested as described by Kumar [27]. Antibiotic sensitivity was determined using Abtex Biologicals discs pre-inoculated with antibiotics or adding 5 µl of prepared antibiotic stocks to a 5 mm piece of filter paper placed on an inoculated NA plate.

Whole-cell fatty acid profiles were determined at Fera Science Ltd Environmental Laboratories (York, UK) according to the methods in Sasser [28], using 48 h grown cultures on tryptone soya broth agar (VWR) supplemented with 2 % (w/v) NaCl. Respiratory quinones and polar lipids were extracted and analysed at DSMZ as described by Tindall [29].

Cells of strain MKS20^T^ were motile, Gram-stain-negative, facultatively anaerobic, 0.5–1.0 µm wide and 1.3–3.0 µm long. Cells form rounded white-beige-pigmented colonies between 1.0–1.5 mm in diameter within 24 h of growth. Optimal growth occurred at 1–3 % (w/v) NaCl (range, 0.5–10.0 %), 28–30 °C (range, 5–37 °C) and at pH 7.0–7.5 (range, pH 6–9.5). Strain MKS20^T^ was oxidase- and catalase-positive, but negative for nitrate reduction and amylase production. Strain MKS20^T^ showed no anaerobic growth on NA with or without NaNO_3_ and also tested negative for nitrate reduction using the API 20E test. According to API 50CH test results, our strain produced acid from selected substrates, indicating fermentative capabilities (Table 1). Cells of strain MKS20^T^ were resistant to ampicillin (2 µg), penicillin (1.5 IU) and streptomycin (10 µg), but susceptible to chloramphenicol (10 µg), erythromycin (10 µg), cefoxitin (30 µg), sulphafurazole (100 µg), tetracycline (10 µg), apramycin (20 µg), carbenicillin (100 µg) and levofloxacin (5 µg). Additional biochemical and physiological characteristics of strain MKS20^T^ and related *Motilimonas* species are summarized in Table 1 and the species description.

The major fatty acids (>10 %) of strain MKS20^T^ were summed feature 3 (C_16 : 1_ ω6*c* and/or C_16 : 1_ ω7*c*) and C_16 : 0_, fitting with the profiles observed for the other species within this genus. The minor fatty acids (<10 %) consisted of summed feature 2 (C_14 : 0_ 3-OH and/or iso-C_16 : 1_), summed feature 8 (C_18 : 1_ ω6*c* and/or C_18 : 1_ ω7*c*), C_14 : 0_, C_18 : 0_ and C_17 : 0_, and showed higher variability across the genus. The fatty acid profile of strain MKS20^T^ differs from other species of *Motilimonas* in their relative proportions of its components as well as the absence of minor fatty acids such as C_12 : 0_ (see Table 2 for the full profile of all strains). The polar lipid profile consisted of phosphatidyglycerol, diphosphatidylglycerol, phosphatidylethanolamine and several unidentified components (one lipid, one aminophospholipid, three aminolipids and four phospholipids; Fig. S1). The presence of three unidentified aminolipids distinguishes strain MKS20^T^ from other species of the genus *Motilimonas*. Strain MKS20^T^ contained the sole respiratory quinone of Q-8, which is in agreement with what is observed for the other species in this genus.

**Table 2.** Mean fatty acid composition of *Motilimonas* strains All strains were grown for 48 h on tryptone soya broth agar supplemented with 2 % (w/v) NaCl. Values are percentages of total fatty acids.

Fatty acid	MKS20^T^	*M. eburnea* YH6^T^	*M. pumila* PLHSC7-2^T^
Summed feature 3 (C_16 : 1_ ω6*c* and/or C_16 : 1_ ω7*c*)	48.0	42.4	36.3
C_12 : 0_	–	2.8	3.0
C_12 : 0_ 3-OH	–	–	0.3
C_14 : 0_	1.2	3.4	2.5
Summed feature 2 (C_14 : 0_ 3-OH and/or iso-C_16 : 1_)	7.0	4.3	5.3
C_16 : 0_	36.0	36.0	47.1
C_16 : 1_ ω5*c*	–	0.6	–
Summed feature 8 (C_18 : 1_ ω6*c* and/or C_18 : 1_ ω7*c*)	6.3	9.6	4.4
C_18 : 0_	0.6	0.5	1.1
C_17 : 0_	0.9	0.4	–

Strain MKS20^T^ represents the first described member of the genus *Motilimonas* isolated from a non-marine environment, thus extending its ecological reach.

The results from the genotypic, physiological and chemotaxonomic comparison show that strain MKS20^T^ can be distinguished from other recognized species of the genus *Motilimonas*. We therefore propose that the strain represents a novel species, with the name *Motilimonas cestriensis* sp. nov.

## Description of *Motilimonas cestriensis* sp. nov.

*Motilimonas cestriensis* (ces.tri.en’sis, M.L. fem, adj. *cestriensis*, from Chester/Cheshire, referring to the Cheshire salt district, the place of isolation).

Cells are facultatively anaerobic, Gram-stain-negative, motile rods (0.5–1.0×1.3–3.0 µm). Colonies are white-beige-pigmented, circular, rounded, moderately opaque and 1–1.5 mm in diameter when grown on NA at 30 °C for 24 h. Optimal growth at 28–30 °C (range, 5–37 °C), 1–3 % (w/v) NaCl (range, 0.5–10 .0%) and at pH 7.0–7.5 (range, pH 6.0–9.5). Cells are amylase-, lipase- (C14) and urease-negative, but positive for oxidase, catalase, esterase (C4), esterase lipase (C8), acid phosphatase, alkaline phosphatase, α-glucosidase and *N*-acetyl-β-glucosaminidase. Negative for the Voges–Proskauer test, nitrate reduction, indole production and production of H_2_S. Acid is produced from glycerol, d-mannitol, trehalose, d-glucose, d-fructose and d-mannose, but not from potassium 5-ketogluconate, cellobiose or lactose. Cells are able to oxidize dextrin, maltose, trehalose, gentiobiose, turanose cellobiose, d-fructose, *N*-acetyl-β-d-mannosamine, inosine, d-sorbitol, d-mannitol, glycerol, l-alanine, l-glutamic acid, l-serine, methyl pyruvate, l-malic acid, bromo-succinic acid, Tween 40, acetic acid and l-aspartic acid, but not propionic acid, l-histidine, glucuronamide, methyl β-d-glucoside, d-serine, or sucrose.

The major fatty acids (>10 %) are summed feature 3 (C_16 : 1_ ω6*c* and/or C_16 : 1_ ω7*c*) and C_16 : 0_. The polar lipid profile consists of phosphatidyglycerol, diphosphatidylglycerol, phosphatidylethanolamine and several unidentified components (one lipid, one aminophospholipid, three aminolipids and four phospholipids). Q-8 is the sole respiratory quinone. The genomic DNA G+C content of the type strain is 44.2 mol%.

The type strain, MKS20^T^ (=DSM 109936^T^=MCCC 1K04071^T^), was isolated from an inland brine spring in Anderton, within the Cheshire salt district (UK).

## Supplementary Data

Supplementary material 1
