# Supplementary material for: Motilimonas cestriensis sp. nov., isolated from an inland brine spring in Northern England
Source: Int J Syst Evol Microbiol. 2021 Mar 19;71(3):004763. doi: 10.1099/ijsem.0.004763 (PMC8375427; doi:10.1099/ijsem.0.004763)
Supplement: Supplementary material 1 [file ijsem-71-4763-s001.pdf]

International Journal of Systematic and Evolutionary Microbiology

Motilimonas cestriensis sp. nov., Isolated from an Inland Brine Spring in Northern England

Matthew Kelbrick[1, 2], Raeid M. M. Abed[3], André Antunes[4,*]

[1]Biology Department, Edge Hill University, Ormskirk, United Kingdom

[2]Department of Evolution, Ecology and Behaviour, Institute of Infection, Veterinary & Ecological Sciences, University of Liverpool, Liverpool, United Kingdom

[3]Biology Department, College of Science, Sultan Qaboos University, Al Khoud, Sultanate of Oman

[4]State Key Laboratory of Lunar and Planetary Sciences, Macau University of Science and Technology (MUST), Macau SAR, China

[*]Correspondence: André Antunes, aglantunes@must.edu.mo

Supplementary Material

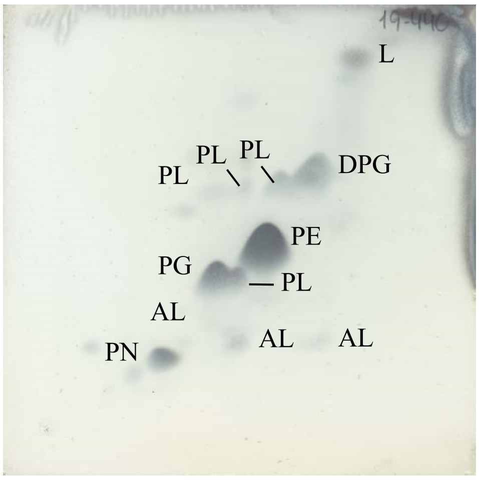

Fig. S1. Polar Lipid profile using two-dimensional liquid chromatography for strain MKS20[T]. L, Lipid; AL, Aminolipid; PL, Phospholipid; PN, Aminophospholipid; PG, Phosphatidylglycerol; PE, Phosphatidylethanolamine; DPG, Diphosphatidylglycerol.

Table S1: Genomic comparison of type species of the genus *Motilimonas*. dDDH, digital DNA-DNA hybridisation; ANI, average nucleotide identity; AAI, average amino-acid identity; 16S, 16S rRNA gene.

		MKS20^T	*M. eburnea* YH6	*M. pumila* PLHSC7-2^T
dDDH	MKS20^T	100 %		
	M. eburnea YH6^T	20.60 %	100 %	
	M. pumila PLHSC7-2^T	25.10 %	24.40 %	100 %
ANI	MKS20^T	100 %		
	M. eburnea YH6^T	79.60 %	100 %	
	M. pumila PLHSC7-2^T	78.86 %	77.28 %	100 %
AAI	MKS20^T	100 %		
	M. eburnea YH6^T	77.43 %	100 %	
	M. pumila PLHSC7-2^T	67.86 %	67.06 %	100 %
16S	MKS20^T	100 %		
	M. eburnea YH6^T	98.30 %	100 %	
	M. pumila PLHSC7-2^T	96.62 %	96.19 %	100 %
